# The distribution and transitions of physicians in Japan: a 1974–2004 retrospective cohort study

**DOI:** 10.1186/1478-4491-7-73

**Published:** 2009-08-14

**Authors:** Hiroo Ide, Soichi Koike, Tomoko Kodama, Hideo Yasunaga, Tomoaki Imamura

**Affiliations:** 1 Department of Planning, Information and Management, The University of Tokyo Hospital, Tokyo, Japan; 2 Department of Global Health and Population, Harvard School of Public Health, Boston, Massachusetts, USA; 3 Department of Policy Sciences, National Institute of Public Health, Saitama, Japan; 4 Department of Health Management and Policy, Graduate School of Medicine, The University of Tokyo, Tokyo, Japan; 5 Department of Public Health, Health Management and Policy, Nara Medical University, Nara, Japan

## Abstract

**Background:**

In Japan, physicians freely choose their specialty and workplace, because to date there is no management system to ensure a balanced distribution of physicians. Physicians in Japan start their careers in hospitals, then become specialists, and then gradually leave hospitals to work in private clinics and take on primary care roles in their specialty fields. The present study aimed to analyse national trends in the distribution and career transitions of physicians among types of facilities and specialties over a 30-year period.

**Methods:**

We obtained an electronic file containing physician registration data from the Survey of Physicians, Dentists and Pharmacists. Descriptive statistics and data on movement between facilities (hospitals and clinics) for all physicians from 1974, 1984, 1994 and 2004 were analysed. Descriptive statistics for the groups of physicians who graduated in 1970, 1980 and 1990 were also analysed, and we examined these groups over time to evaluate their changes of occupation and specialty.

**Results:**

The number of physicians per 100 000 population was 113 in 1974, and rose to 212 by 2004. The number of physicians working in hospitals increased more than threefold. In Japan, while almost all physicians choose hospital-based positions at the beginning of their career, around 20% of physicians withdrew from hospitals within 10 years, and this trend of leaving hospitals was similar among generations. Physicians who graduated in 1980 and registered in general surgery, cardiovascular surgery or paediatric surgery were 10 times more likely to change their specialty, compared with those who registered in internal medicine. More than half of the physicians who registered in 1970 had changed their specialties within a period of 30 years.

**Conclusion:**

The government should focus primarily on changing the physician fee schedule, with careful consideration of the balance between office-based physicians and hospital-based physicians and among specialties. To implement effective policies in managing health care human resources, policy-makers should also pay attention to continuously monitoring physicians' practising status and career motivations; and national consensus is needed regarding the number of physicians required in each type of facility and specialty as well as region.

## Background

A balanced health workforce is a key factor in strengthening health care systems. Policy-makers should aim to "get the right workers with the right skills in the right place doing the right things" [1]. The geographical distribution of physicians in several developed countries has been analysed in previous studies [2-4]. However, more studies are needed in order to implement effective human resource policies [5].

In Japan, in making their career choices, physicians generally consider the combined factors of specialty and working facility. Almost all newly-graduated Japanese physicians become hospital-based physicians (HP), who are employed as full-time workers by hospitals; there they are given training to become specialists. After working for several years, these physicians may resign from their hospital positions and become self-employed, office-based physicians (OP). In this regard, OPs are not originally trained as general practitioners.

OPs see only primary care patients in their private offices. They generally see patients with diseases and symptoms that fall within their specialty area. There are few primary care physicians who have been trained like those in the United Kingdom, where primary care is recognized as a specialty, and primary care physicians (general practitioners) are trained through a system that covers all primary care fields. In addition, Japan does not have a compulsory distribution system to balance the supply of physicians around the country [3]. Since the late 1980s, administrative regulation has prohibited the establishment of new hospitals, but the establishment of new clinics and the selection of specialties are carried out according to individual physicians' preferences.

However, Japan is now facing a maldistribution of physicians between hospitals and private clinics [6,7]. Studies that elucidate the dynamics of physicians' career choices among specialties and facilities are needed as a basis for instituting appropriate human resource policies. Such studies can also be applicable in countries facing a similar situation to Japan's, where physicians are allowed to move and work freely and do not have a strict specialty certification system.

In Japan, the official survey of physician registration is the Survey of Physicians, Dentists and Pharmacists (SPDP), conducted once every two years. Through this survey, all physicians are legally obliged to report their employment status, including their workplace and position, to the Ministry of Health, Labour and Welfare (MHLW). For our study, we used this retrospective data to analyse national trends in the distribution and employment transitions of physicians over a recent 30-year period.

## Methods

### Data collection

We obtained from the MHLW an electronic file containing all the data from the SPDP from 1972 to 2004. The items reported in the SPDP include year of registration, medical license registration number, year of birth, gender, workplace address, and occupation type and specialty. The data did not include any personal information by which an individual could be identified. Japan's Privacy Act defines personal information as any information that any other entities can use to identify a person or can use to do so in combination with other sources of information.

For the present study, we organized the longitudinal data for all physicians by retrieving their unique registration numbers, which are given sequentially to all physicians who pass the national examination. Then we performed data cleansing to make the collection of data complete, and in total 4 024 916 items of data (for 374 804 physicians) were obtained. The notification rate for each implementation of the SPDP was approximately 90% [8].

### Descriptive statistics

From the survey data for 1974, 1984, 1994 and 2004, we determined the total numbers of all physicians surveyed, along with the numbers of physicians per 100 000 population, the percentages of physicians working at hospitals, the percentages of female physicians, the percentages of physicians working in rural areas and the average ages of physicians. The national population in these years was obtained by referring to the Japan Population Census and the Population Estimate.

The group of physicians who graduated in 1970 was defined as the class of 1970. The same was done for the class of 1980 and of 1990. Some statistics, as outlined below, were calculated starting from the physicians' fifth year of experience.

In examining some career aspects, it is appropriate to analyse physicians' choices from the time when they became certified in a specialty, because years of practice and case experience are necessary before physicians can become certified. However, the SPDP does not record specialty certification status, and physicians are allowed to present themselves as specialists in any field, even more than one field, according to the Physicians Law, on the sole condition that they have an active license. In our assessment of certification status, we examined physicians' career behavior from their fifth year of practice because we assumed that they had chosen their specialties by that time.

For each of the three graduating classes, we calculated the number of physicians in their fifth year of experience, percentage of female physicians in their fifth year of experience, average age at first registration, percentage of physicians working in a specialty and medical facility in their fifth year of experience, average lifetime frequency of specialty changes since their fifth year of experience, and percentage of physicians changing specialties more than once. A comparison of average values between two classes was performed by means of a t-test, and a comparison of rates between two classes was performed by means of a Chi-square test.

### Analysis of movement from hospital-based to office-based practice

The numbers of physicians registered as HPs in 1974, 1984, 1994 and 2004 were defined as N1, N2, N3 and N4, respectively. In N1, the number of HPs who withdrew from hospital work between 1975 and 1984 was defined as R1, and the number of HPs who remained in hospital work during that period was defined as C1. The number of new graduates who began to work in hospitals between 1975 and 1984 was defined as P1. In the same way, between 1984 and 1993, and 1994 and 2003, the numbers of HPs who withdrew from hospital work were defined as R2 and R3, respectively; the numbers of HPs who remained in hospital work were defined as C2 and C3, respectively; and the numbers of physicians who began to work in hospitals were defined as P2 and P3, respectively.

N1 = R1 + C1, N2 = C1 + P1

N2 = R2 + C2, N3 = C2 + P2

N3 = R3 + C3, N4 = C3 + P3

The number of physicians registered as OPs in 1974, 1984, 1994 and 2004 were defined as n1, n2, n3 and n4, respectively. In n1, the number of those who retired as OPs between 1975 and 1984 was defined as r1, and the number of those who continued as OPs during that period was defined as c1. The number of those who newly started work as OPs between 1975 and 1984 was defined as p1. In the same way, r2, r3, c2, c3, p2 and p3 were defined.

n1 = r1 + c1, n2 = c1 + p1

n2 = r2 + c2, n3 = c2 + p2

n3 = r3 + c3, n4 = c3 + p3

These variables were identified to analyze the career movement of HPs and OPs.

### Follow-up research on leaving rates of HPs

For each of the classes of 1970, 1980 and 1990, physicians who worked in hospitals in their fifth year of experience were defined, and the numbers of those who later withdrew from hospital work were noted. A log-rank test was used to compare differences in leaving rates.

### Evaluation of the factors influencing specialty changes

For each of the classes of 1970, 1980 and 1990, a multivariate logistic regression analysis was performed to elucidate the factors influencing specialty changes. (If a physician changed his/her specialty after his/her fifth year of experience, the value of the dependent variable was 1.) The independent variables were gender, age at first registration, specialty in their fifth year of experience, working area (urban, rural and intermediate areas) in their fifth year of experience and work facility in their fifth year of experience. All statistical analyses were performed by means of the statistical software SPSS, version 13.0 (SPSS, Chicago, United States). A *p*-value of less than 0.05 was considered to be significant.

## Results

### Descriptive statistics

The total number of physicians doubled during the 30-year study period. Table 1 shows the descriptive data for each measure from 1974, 1984, 1994 and 2004. The number of physicians per 100 000 population was 113 in 1974; by 2004 this had risen to 212, indicating an increase of 87%. Compared with 1974, the percentage of physicians working in rural areas (11%) decreased by 2004, although the actual number of physicians working in those areas substantially increased. The percentage of female physicians (17%) increased significantly (*p *< 0.01).

**Table 1:** Descriptive statistics

		**1974**	**1984**	**1994**	**2004**
Number of physicians	Total	125 249	178 197	227 775	270 353
	
	Hospitals	54 005	100 018	142 309	170 386
	
	Clinics	65 099	70 662	76 596	92 982

Number per 100 000 population		113	148	182	212

Working at hospitals (%)		43	56	62	63

Female (%)		9	10	13	17

Working in rural areas (%)		14	14	13	11

Average age (± SD)	Total	47.6 (14.0)	46.9 (14.9)	46.7 (15.4)	47.8 (15.2)
	
	Hospital-based physicians	40.4 (14.5)	39.4 (12.5)	40.2 (13.2)	42.0 (12.6)
	
	Office-based physicians	53.2 (10.4)	57.0 (11.2)	58.1 (12.3)	57.5 (13.8)

In 2004, the average age of OPs was 57.5 years, which was significantly higher than that of HPs (42.0 years) (*p *< 0.01). The number of physicians in hospitals as well as those in clinics increased during the study period. However, the proportion of physicians working in hospitals rose to 63% by 2004 from 43% in 1974 (Table 1).

The average ages at first registration for the classes of 1970, 1980 and 1990 were 26.3, 26.7 and 26.7, respectively, indicating that the latter two were significantly higher than the former (*p *< 0.01). In all the classes, over 90% of physicians worked in hospitals in their fifth year of experience. The average frequencies of specialty changes for the classes of 1970, 1980 and 1990 were 1.5, 0.8 and 0.4, respectively. Among the class of 1970, 53% of physicians changed their specialty more than once during the course of their career (Table 2).

**Table 2:** Descriptive statistics of the classes of 1970, 1980 and 1990

		**Class of 1970**	**Class of 1980**	**Class of 1990**
Number of physicians in their fifth year of experience		2706	6326	6994

Females in their fifth year of experience (%)		9	11	18

Average age at first registration (± SD)		26.3 (2.2)	26.7 (2.7)	26.7 (2.7)

Work facility in their fifth year of experience (%)	Clinics	5	4	3
	
	Hospitals	91	93	94
	
	Others	4	4	3

Average frequency of lifetime specialty changes (± SD)		1.5 (2.0)	0.8 (1.3)	0.4 (0.8)

Percentage of physicians changing specialties more than once (%)		53	38	27

### Analysis of the movement of HPs and OPs

**Figure 1:**
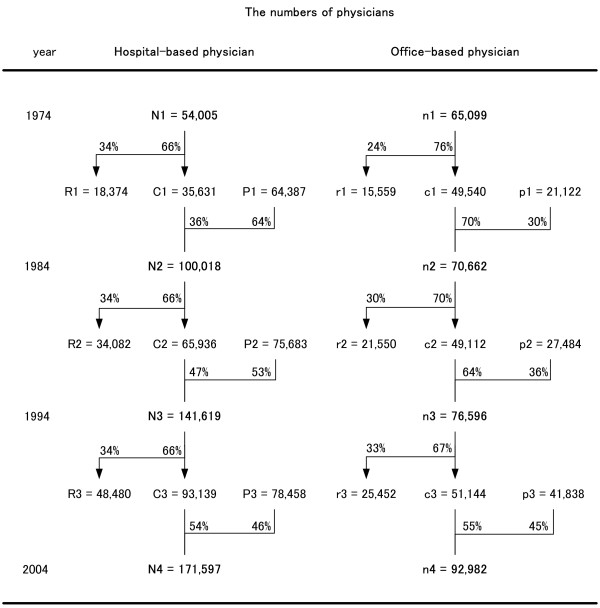
**The career movement of hospital-based and office-based physicians**. N1, the number of physicians working in hospitals in 1974; N2, the number in 1984; N3, the number in 1994; N4, the number in 2004. R1, the number of physicians who withdrew from hospitals between 1975 and 1984; R2, between 1985 and 1994; R3, between 1995 and 2004. C1, the number of physicians who remained working in hospitals from 1974; C2, from 1984; C3, from 1994. P1, the number of new physicians who began to work in hospitals between 1975 and 1984; F2, the number between 1985 and 1994; F3, the number between 1995 and 2004. n1, the number of physicians working in clinics in 1974; n2, the number in 1984; n3, the number in 1994; n4, the number in 2004. r1, the number of physicians who retired as office-based physicians between 1975 and 1984; r2, between 1985 and 1994; r3, between 1995 and 2004. c1, the number of physicians who continued as office-based physicians from 1974; c2, from 1984; c3, from 1994. p1, the number of new physicians who began to work as office-based physicians between 1975 and 1984; p2, the number between 1985 and 1994; p3, the number between 1995 and 2004.

Figure 1 shows the trends in the numbers of HPs and OPs. The number of HPs increased more than threefold between 1974 and 2004, and exceeded the number of OPs during 1974 and 1984. Even though the total numbers of HPs (N1, N2, N3 and N4) changed, the percentages of physicians who withdrew from hospitals remained stable (36%).

### Follow-up research on leaving rates of HPs

**Figure 2:**
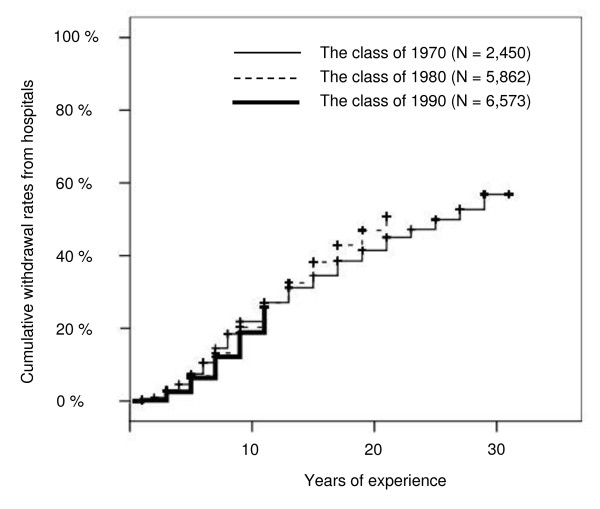
**Cumulative withdrawal rates of hospital-based physicians from hospital**.

Figure 2 shows the cumulative rates of HPs who withdrew from hospital work in each of the classes of 1970, 1980 and 1990. The numbers of HPsin the classes of 1970, 1980 and 1990 were 2450, 5862 and 6573, respectively. Among the class of 1970, 57% of physicians who worked at hospitals in their fifth year of experience left their hospital positions within 30 years. While a log rank test showed a statistically significant difference in leaving rates of HPs among the classes (*p *< 0.01), around 20% (19% to 22%) of all physicians withdrew from hospital work within 10 years, and the trends in leaving rates were similar between the classes.

### Evaluation of the frequency of and factors influencing specialty changes

Among the class of 1980, physicians who registered their specialty as general surgery (odds ratio (OR, 7.2), cardiovascular surgery (OR, 11.6), or paediatric surgery (OR, 11.3) had a higher OR for changing their specialty, compared with those who registered in internal medicine (base category). On the other hand, physicians who registered their specialties as ophthalmology (OR, 0.4) or otolaryngology (OR, 0.6) showed a lower OR compared with those in internal medicine (Table 3). Female physicians were 1.3 to 1.5 times more likely to change their specialty. The age at first registration and working area (except, among the class of 1970, those in intermediate areas) did not predict physicians' specialty changes.

**Table 3:** Results of the logistic regression analysis for specialty changes in the classes of 1970, 1980 and 1990

		**Odds ratios (95% confidence interval)**		
		
		Class of 1970 N = 2,706	Class of 1980 N = 6,326	Class of 1990 N = 6,994
Sex (base category; men)		1.5 (1.3 – 1.7)	1.4 (1.3 – 1.5)*	1.3 (1.2 – 1.4)*
Age at first registration		ns	ns	ns
Specialty	Paediatrics	1.8 (1.6 – 2.0)*	2.1 (2.0 – 2.2)*	ns
(base category; internal medicine)	General surgery	11.2 (11.1 – 11.3)*	7.2 (7.1 – 7.3)*	3.7 (3.6 – 3.8)*
	Neurosurgery	3.9 (3.6 – 4.2)*	2.5 (2.3 – 2.7)*	ns
	Respiratory surgery		na	17.1 (16.6 – 17.6)*
	Cardiovascular surgery		11.6 (11.2 – 12.0)*	5.6 (5.4 – 5.8)*
	Paediatric surgery		11.3 (10.7 – 11.9)*	8.3 (7.7 – 8.9)*
	Orthopaedics	1.8 (1.7 – 1.9)*	1.4 (1.3 – 1.5)*	ns
	Plastic surgery		3.9 (3.6 – 4.2)*	ns
	Obstetrics and gynaecology	ns	ns	0.3 (0.1 – 0.5)*
	Ophthalmology	ns	0.4 (0.2 – 0.6)*	0.2 (0.0 – 0.4)*
	Otolaryngology	0.5 (0.2 – 0.8)	0.6 (0.4 – 0.8)*	0.4 (0.2 – 0.6)*
	Dermatology	ns	ns	0.6 (0.4 – 0.8)
	Urology	ns	2.0 (1.8 – 2.2)*	ns
	Rehabilitation			7.9 (7.2 – 8.6)*
	Radiology	10.7 (10.3 – 11.1)*	4.8 (4.6 – 5.0)*	1.8 (1.6 – 2)*
	Anaesthesiology	8.0 (7.6 – 8.4)*	2.9 (2.8 – 3.0)*	1.7 (1.6 – 1.8)*
	Psychiatrics	2.7 (2.5 – 2.9)*	1.6 (1.5 – 1.7)*	1.1 (1.0 – 1.2)
	Others	69.4 (68.4 – 70.4)*	19.6 (19.3 – 19.9)*	13.9 (13.7 – 14.1)*
Working area	Intermediate area	0.8 (0.7 – 0.9)	ns	ns
(base category; urban area)	Rural area	ns	ns	ns
Work facility	Hospitals	0.7 (0.5 – 0.9)	0.6 (0.5 – 0.7)*	0.5 (0.3 – 0.7)*
(base category; clinics)	Others	0.1 (-1.0 – 1.2)	0.1 (-0.3 – 0.5)*	0.3 (0.0 – 0.6)*

* p < 0.01

ns: not significant; na: not available

R2 for the analyses of the classes of 1970, 1980 and 1990 were 0.24, 019 and 0.17, respectively.

## Discussion

### Transitions in the physician workforce

The total number of physicians in Japan dramatically increased between 1974 and 2004. In particular, the number of HPs increased more than threefold by 2004, and the ratio of HPs to OPs was reversed compared with that of 1974. One factor influencing the dynamics of physicians in Japan is that the number of students enrolling in medical schools doubled during the 1970s. The number of hospitals also increased until 1986, when the government placed restrictions on the establishment of new hospitals. Additionally, the increase in the ratio of HPs to OPs suggests that Japan's health care system, in terms of its physician workforce, has shifted its focus from primary care to specialty care over this recent 30-year period.

### Why do physicians change workplaces?

Even though the number and percentage of HPs rose, our results show that the rates of career movement from hospitals (specialty care) to clinics (primary care) have generally been stable for many years.

Two alternative reasons for physicians' career changes can be considered. First, salary considerations may motivate HPs to leave hospital work. HP salaries are relatively low, compared with OP salaries. As of 2008, although there were 8807 hospitals (employing about two thirds of all physicians) and 99 581 clinics with fewer than 20 inpatient beds in Japan, hospitals provide around one third of all outpatient services and 94% of inpatient services [9,10]. This indicates that the separate roles of hospitals and clinics are not well defined in the Japanese health care system, and that a substantial responsibility of hospitals is to provide outpatient care. Regarding the difference in income between OPs and HPs, this situation has a historical context, and thus it is politically difficult to work towards a salary balance between OPs and HPs [11,12]. Recently, much effort and many opinions have been directed at working towards a balance, but the actual fee schedule has not yet been modified.

Second, as a physician gets older and feels the burden of long hours and being on call, he/she may choose to leave the hospitals and begin to work as a primary care physician in a private office. In hospitals, physicians are usually required to perform operations and invasive examinations. Such intensive job burdens can affect physicians' willingness to continue working. Although previous studies have showed that physicians' intentions to leave differ among specialties, physicians working in high-risk specialties are less satisfied [13,14] and more inclined to change jobs [15].

In Japan, HPs' working conditions, in terms of working hours, job stress and risk of lawsuits, are generally more intense than those of OPs. Therefore, as HPs age, more of them gradually decide to leave hospitals, and this behaviour does not change with the generation. However, a recent estimate showed that the number of OPs will increase by 37.6% from 2004 to 2016 [16]. Physicians' career behaviour has been relatively stable in the Japanese system, but new factors may gradually cause it to change. For example, the increase in female physicians, a general preference for a more controllable lifestyle [17,18] and other generation/cohort effects may be found to be influential.

Contrarily, older physicians can continue to practise longer in the Japanese system, and many continue until they are in their 70s. A probable reason for this is that older physicians working as OPs can attend to outpatients without having to engage in heavier aspects of hospital practice such as invasive examinations, operations and night shifts. Moreover, the monetary incentive resulting from the skewed fee schedule (between hospital and office practices), which is favourable for OPs, encourages them to remain in practice longer.

### Why do physicians leave high-workload specialties?

According to our results, the range of available specialist physicians in Japan is threatened. Among the class of 1970, physicians changed their specialty an average of 1.5 times after more than 30 years of experience, with 53% of physicians changing at least once. In addition to this, physicians who initially registered in high-workload specialties such as general surgery, cardiovascular surgery or paediatric surgery were about 10 times more likely to change their specialty, compared with those who registered for internal medicine. Thus the experience and skills of specialty physicians in Japan may be lacking, and this could affect the health status of the public. However, many indices show that Japanese people's current health status is better than that of citizens of many other countries [19,20].

The difference in the fee schedule between different specialties is probably another reason for inter-specialty changes. The charges for operations are not so high under the uniform fee schedule in Japan that hospitals can pay enough salary for surgeons. If more physicians are required in heavy workload specialties such as surgery, financial incentives for practising in such specialties should be offered.

Although providing financial incentives for surgeons is considered to be an essential way to solve these problems, we should carefully consider the potential side effects. In Japan, the government can neither allocate more of its budget to health care nor increase taxes and health insurance premiums. Japan is a country where tax and social insurance rates for income are the lowest among OECD countries [21]. However, the public may not accept an increase to these rates. Thus, setting higher fees for specialists working in high-workload fields requires changes in budget allocations, shifting focus from low-workload to high-workload specialties.

Meanwhile, it is likely that physicians' career behaviours will generally reverse direction as a result of such reallocations. For example, the United States is an example of a country where fees for primary care physicians are low [22]. This fact is not only a problem for current primary care physicians but also a reason why new medical graduates tend not to choose primary care as their specialty [23,24]. A radical fee allocation change in Japan could create the same type of situation.

Meanwhile, it should also be considered why the high rates of physicians leaving high-workload specialties have not harmed the general health status in Japan. First, it may be that the number of physicians has a weak relationship with general health status, although this is still unconfirmed [25]. Second, the number of specialists in Japan is not controlled from the beginning of specialists' training, so there are many more practising specialists than are actually needed. And a rather preferable tendency may be occurring: less competitive specialists leaving their positions. As supporting evidence for this trend, the Japanese Society for Cardiovascular Surgery recently decided that physicians applying to be certified as cardiovascular surgeons must have previously practised in hospitals where there is a minimum number of cardiovascular operations [26], and this may indirectly affect the number of physicians applying for work in cardiovascular surgery.

### Possible impact of changes in initial clinical training system

Japan introduced a new clinical training system in 2004, and this will probably affect physicians' career choices in the future. Before 2004, most new physicians had their initial clinical training at academic hospitals. The curriculum was planned by each academic hospital, with an emphasis on specialty care but not primary care. This was one of the reasons why the government significantly changed the clinical training system in 2004.

Under the new system, physicians are required to experience a clinical rotation in the fields of general internal medicine, general surgery, emergency medicine, paediatrics, obstetrics and gynecology, psychiatry and community medicine [27,28]. When they finish this general postgraduate training, they are allowed to begin specialized training.

Previous to this system change, 55% of new physicians began their careers at non-academic hospitals, and in 2004, 40% began their careers at academic hospitals [16]. Although it is difficult to predict the results of this fundamental change in the clinical training system, its impact will possibly be larger than that of a change in the fee schedule.

### Study limitations

This study has some limitations. First, our data did not directly elucidate physicians' motives in making career decisions, because the SPDP in Japan does not ask physicians about their reasons for changing workplaces, occupations or specialties. In comparison, the American Medical Association Physician Masterfile has more detailed information [29].

In addition to a basic physician tracking system, it would be helpful to policy-makers who are managing the physician workforce in Japan to know physicians' motives and personal characteristics, in order to plan appropriate incentives. For instance, physicians' geographical origins [30-32], strong intentions for particular specialties [31], education [31-34] and test scores [35,36] can affect their career choices. Meanwhile, their job satisfaction is likely correlated with their sense of motivation and satisfaction with their retirement plan [15]. Without evidence on these aspects, governments cannot implement evidence-based policies to address the urgent problems in the health workforce.

Second, there are limitations, other than those we mentioned above, to the use of SPDP data as official statistics for analysing the workforce supply. For example, physicians have to report their status only while their licence is valid. So if a physician dies before cancelling his/her licence, the government is not able to determine why that physician stopped practising: whether because of retirement or death.

Moreover, the SPDP does not have a question item to determine whether a physician works full-time or part-time, and at what workplaces. As a result, our analysis was based on a headcount, but the actual workforce supply could differ from the headcount.

In addition, Japanese specialty certification, which is issued by each specialty's physicians' society, seems to lack rigidity and was introduced relatively recently, compared with those in other developed countries [28]. Even so, in the future, the SPDP should include items regarding certification status.

Third, we were not able to address the consideration of the number of physicians required in hospitals and clinics and in each specialty, because Japan does not have an official estimate indicating such appropriate numbers. Without this estimate, we cannot fully evaluate the physician distribution [37].

## Conclusion

In Japan, the focus of the health care system has changed from primary to specialty care over the 30-year period from 1974 to 2004. Although the movement from hospitals to clinics is stable among generations, more than half of the physicians who registered in 1974 changed their specialties, and physicians working in high-workload specialties were much more inclined to change their specialties.

Even while physicians' career behaviours could be partly explained by certain aspects of human nature, and while other factors of the clinical training system and certification system also should be considered, the government should focus primarily on changing the physician fee schedule. This should be done with careful consideration of the balance between OPs and HPs and among specialties. To implement effective policies for health care human resources, policy-makers should pay attention to continuously monitoring physicians' practicing status and career motivations, and national consensus is needed regarding how many physicians are required at each type of facility and specialty as well as region.

## Competing interests

The authors declare that they have no competing interests.

## Authors' contributions

All the authors conceived the study and jointly designed and conducted it. HI and HY analysed the data and all the authors interpreted the results. HI and HY drafted the manuscript, and all the authors revised it and approved the final version. All the authors take public responsibility for the content of the manuscript.
